# Changing Pattern of Hepatitis A Virus Epidemiology in an Area of High Endemicity

**DOI:** 10.5812/hepatmon.5940

**Published:** 2012-06-30

**Authors:** Marcello Campagna, Andrea Siddu, Angelo Meloni, Claudia Basciu, Luigi Ferrai, Alessandro Pettinau, Cristiana Cardia, Giuseppina Masia, Rosa Cristina Coppola

**Affiliations:** 1 Department of Public Health, University of Cagliari, Cagliari, Italy

**Keywords:** Hepatitis A, Epidemiology, Immunity, Herd, Preventive Measures

## Abstract

**Background:**

Continuous assessment of hepatitis A virus (HAV) seroepidemiology is a useful tool to control the risk of infection.

**Objectives:**

This study aimed to evaluate the changing patterns of anti-HAV seroprevalence in a population,which isgenerally considered to be anarea ofhigh endemicity.

**Patients and Methods:**

Overall, the results of 3349 sera collected during the period 2005-2008 from patients attending the University Hospital of Cagliari, Italy were studied; their mean age was 52.7 years, (s + 16.22). Patients with liver disease were excluded from the study. Age specific seroprevalence results were compared with those observed in similar previous studies carried out in the same area.

**Results:**

The overall prevalence of anti-HAV was 74.6% with consistently lower values in subjects younger than 40 years (17.5%; P < 0.0001) particularly in those under 30 years of age (8.9%, CI 5.8-11.9). A significant declining trend in age specific seroprevalence has been foundin people under 30 years;61% in 1988, 33% in 1995 and 8.9% in 2005-2008.

**Conclusions:**

Our findings show that a significant decline inherd immunity has occurred in the last 20 years as a consequence of lower HAV circulation due to improvementsin socio-economical and hygienic conditions. Adolescents and young adults are becoming increasingly susceptible to HAV infections, as recent outbreaks of acute HAV hepatitis have occurred. Persistent environmental monitoring and the implementation of prevention measures must be considered in order to contain the risk related to this epidemiological shift.

## 1. Background

Hepatitis A is generally an acute, self-limiting liver infection transmitted through the faecal-oral route by a picornavirus, the Hepatitis A Virus (HAV), which causes 10 million infections worldwide each year[1][2]. The clinical severity of the HAV infection varies from an asymptomatic infection to a fulminant fatal disease [3][4] and age is the major factor that inﬂuences the clinical course of the primary HAV infection; it is symptomatic in only 4–16% of children compared to 75–95% of adults [4]. The degree of endemicity is closely related tothe prevailing hygiene and sanitary conditions, socio-economic level and other development indicators [5]. In recent decades Italy has experienced a declining trend inHAV epidemiology [6], this is probably related to improvementsin health and sanitary conditions, which have been responsible for the progressive decline in infection rates among children under 14 years of age and for a major shift towards the highest incidence in susceptible teenagers and young adults [7]. The integrated epidemiological system for acute viral hepatitis surveillance (SEIEVA) reports that the incidence of HAV has declined from 10/100000 in 1985 to 3.6/100000 in 2004, and to 1.1/100000 in 2010, with an increase during 1996-1997 corresponding to a large outbreak which occurred in two regions of southern Italy (Puglia and Campania). Epidemiological patterns vary among the different regions within Italy, with low endemicity areas in the central and northern regions and intermediate endemicity in the southern and insular regions. The most frequently reported risk factor is the consumption of contaminated seafood, in particularraw or partially cooked shellfish [8][9][10][11].

## 2. Objectives

The aim of the present study was to assess the anti-HAV seroprevalence rates in a sample of population in Southern Sardinia, a major island of Italy, to compare the pattern of immunity with those reported by studies carried out in the same area during the last 20 years, to compare our findings to seroprevalence data from Italy and to determine the most appropriate preventive measures to control and manage the risk of HAV infection.

## 3. Patients and Methods

The results of anti-HAV tests were retrospectively collected from 3349 patients attending the University Hospital of Cagliari, Italy. All subjects affected by or suspected of hepatic disease were excluded from the study in order to avoid possible selection bias resulting in limitations in statistical inference. For the purposesof thisstudy, results were collected anonymously and only the age and gender of subjects and sample date were recorded.

The samples were tested for anti-HAV IgG by a commercially available MicroparticleEnzyme Immunoassay (MEIA; AxSYM-HAVAB 2.0, ABBOTT).All specimens were tested for anti-HAV-IgG: 135 of them (4%) had equivocal analytical results and were excluded; they were equally distributed in the age classes. Overall, age-specific and gender seroprevalence rates were computed. In order to compare results with previous prevalence rates, data from studies carried out in 1988 [12], 1995 [13] and 1999-2000 [14] were considered.

The statistical analysis was performed using a SPSS version 10 program. In order to assess differences in the seroprevalence rates a chi-squared test was used, a significant difference was consideredwhen P < 0.05. A95% confidence interval (CI) and the Poisson confidence interval, were used when the number of observations was lower than ten.

## 4. Results

**Figure 1:**
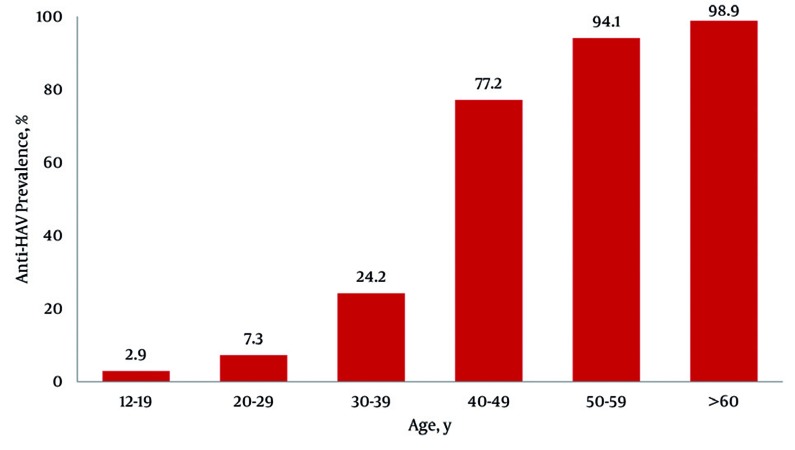
Age Specific Prevalence of Anti-HAV in 3214 Subjects Included in the Study

**Figure 2:**
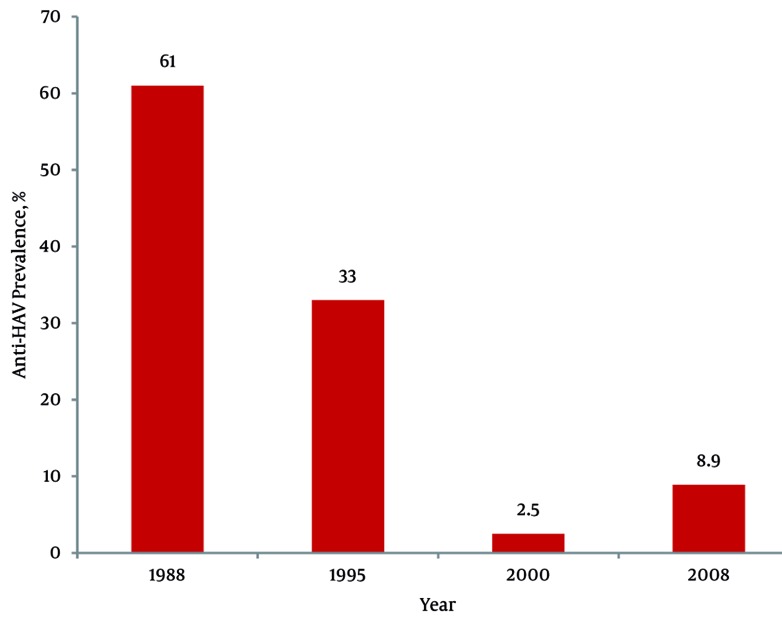
Anti-HAV Prevalence Rates in Subjects Aged 18-30 YearsInvestigated over 20 Years. Age Range of Subjects Studied in 2000 was 18-28 Years.

After the exclusion of the subjects with equivocal analytical results 3214 subjects (1657 males and 1557 females) were included in the study; their distribution according to age and gender is reported in Table 1. Out of the 3214 subjects, 2398 (74.6%; 95% CI: 73,1-76,1) were anti-HAV IgG positive. Age specific prevalence in the study population is reported in Figure 1. The seroprevalence increased progressively with age and differences among the age classes were significant (P < 0.0001); the greatest difference was observed between age classes 30-39 and 40-49-years. The immunity rate was significantly higher in subjects older than 40 years (92%; 95% CI: 91-93) compared with younger subjects (17.5%; 95% CI: 14,8-22) and in older subjects (69.4%; 95% CI 68-70.8) compared with those younger than 30 years (6.8%; 95% CI 5.67.5) (P < 0.0001). The anti-HAV prevalence was significantly higher in males than in females with the greatest difference found in the 20-29 years age class: 11.5% (95% CI: 5,1-17,8) and 4.9% (95% CI: 1,6-8,2) respectively (P < 0.01).Comparing results from previous studies highlights a significant decrease in immunity rates in young adults (P < 0.001) during the two decades as shown in Figure 2.

**Table 1:** Gender Distribution According to Age Classes in 3214 Subjects Enrolled in the Study

**Age Classes, y**	**Males, No.**	**Females, No.**	**Total, No.**
12-19	15	20	35
20-29	96	163	259
30-39	213	242	455
40-49	361	283	644
50-59	326	303	629
> 60	650	542	1192
Total	1661	1553	3214

## 5. Discussion

Overall our data shows a declining trend of herd immunity with regard to HAV infections in the population of South Sardinia during the last 20 years.This dramatic decline was most relevant in young adults and was a result of decreased exposure to HAV during the first years of life. This was probably due to the improvementsin socio-economic and sanitary conditions which have occurred in this area during the past decades and it confirms the changing pattern of immunity, shifting Sardinia from a high to a low HAV endemicity area. Nonetheless, the lower rate of immunity in young adults suggests the persistence of a relevant HAV related risk, due to the marked decline of herd immunity. Our study shows a higher immunity rate compared with seroprevalence values reported in 1999-2000 possibly as the result of an increase in the circulation of HAV in the exposed population from 2000 to 2008.This is also confirmed by reports from thelocal health service regarding outbreaks which occurred in Sardinia in the last few years as a result of raw shellfish consumption, with 94% (31/33) of the cases occuring in young-adults (< 40 years). Several studies have shown thateating raw seafood is the main risk factor for acquiring an HAV infection in Southern Italy [9][10][11] and a previous survey showed that Southern Sardinia’s population regularly eats uncooked shellfish and this was a relevant factorin a proportion of cases (34.7%) [14]. Moreover, some authors have suggested that young adults are exposed more of ten than older people to other well-known risk factors of HAV infection such as; use of intravenous drugs, occupational exposure, homosexual practices and multiple and occasional sexual contacts [15][16]. Paradoxically, our results confirm that as HAVinfections become less common, the risk of new infections shifts from children to adults, and the risk of acute,clinically severe hepatitis A increases [17]. Our study shows ahigher overall rate of immune subjects in Sardinia compared to the rest of Italy. However, this may be due to the higher proportion of subjects older than 40 years in our study population. In fact, considering immunity rates by age class, we observed a higher anti-HAV seroprevalence in the over 40 years age class and a significantly lower seroprevalence rate in subjects younger than 40 years (especially in the 20-29 and 30-39 age class) compared to the rest of Italy and the majority of other European countries [18].

This different seroprevalence picture suggests the need to adopt tailored preventive strategies based on specific risk assessments. In order to reduce the risk of HAV infections in an epidemiological picture characterized by high rates of susceptible subjects among adolescents and young adults, vaccination of household contacts of sporadic cases and vaccination of individuals at higher risk of infection or at risk of complications of HAV hepatitis,in particular those individuals affected by underlying liver disease, represents the main preventive measure. In Italy the current National Vaccination Plan 2005-2008 [19] recommends vaccination against HAV only for specific population groups (travellers to endemic areas, drug users, men who have sex with men (MSM), soldiers, sewage workers, patients presenting with liver disease, recipients of liver transplants and HAV-negative haemophiliacs) [19][20]. In Puglia, a region of Southern Italy, where large epidemics of hepatitis A occurred in the middle 90’s, a mass, free of charge mass vaccination program for newborns (15-18 months) and adolescents (12 years) was introduced in 1998, as part of the routine immunisation schedule [21]. Actually, the current epidemiological situation does not suggest the need for mass vaccination of newborns and adolescents either in Sardinia or in the rest of Italy, and a vaccination program for at risk groups would be more suitable for the control of this virus in young adults [22].

However, in Sardinia, the very low herd immunity picture suggests the need to implement educational campaigns for subjects at higher risk and about dietary habits (to eat only cooked shellfish) as well as the implementation of controls by the local health services regardingsanitation when harvesting shellfish and outlets, in order to avoid this source of infection. Moreover, despite improvementsin hygiene conditions, dietary habits and higher standards of agriculture and manufacturing, the HAV risk associated with importing food from countries with lower standards of environmental hygiene and higher levels of HAV, endemicityremains high. In a global economy fruits and vegetables imported from developing countries can pose a serious risk of an HAV outbreak in a population with no herd immunity, thus allowing the spread of HAV infections from endemic to nonendemic areas [17].

Our study has some strengths and limitations. The main limitation is whether or not the study population is representative of the Southern Sardinia population. In this regard, a possible limitation to our study sample could be that we did not include subpopulations with a high prevalence of HAV infection (i.e., prison inmates and residents of institutions). Likewise, travellers, drug users, people of low socio-economic status and people with health problems may have been overly represented, since a significant rate of serum samples were taken from individuals who received care in hospital or for specific screening. Despite these limitations, the anti-HAV distribution that we observed probably reflects the overall picture of prevalence in the different age groups of Southern Sardinia. In this scenario, seroprevalence studies can provide the most accurate picture of the circulation of HAV in a given population and represent the most appropriate tool for risk assessment. It is in fact suggested that routine surveillance of HAV infection, based solely on the reporting of symptomatic cases who seek medical care, could underestimate the risk [18].
